# iNPH—the mystery resolving

**DOI:** 10.15252/emmm.202013720

**Published:** 2021-02-08

**Authors:** Ville Leinonen, Teemu Kuulasmaa, Mikko Hiltunen

**Affiliations:** ^1^ Institute of Clinical Medicine Neurosurgery University of Eastern Finland Kuopio Finland; ^2^ Department of Neurosurgery Kuopio University Hospital Kuopio Finland; ^3^ Institute of Biomedicine University of Eastern Finland Kuopio Finland

**Keywords:** Genetics, Gene Therapy & Genetic Disease, Neuroscience

## Abstract

Idiopathic normal pressure hydrocephalus (iNPH) is characterized clinically by degradation of gait, cognition, and urinary continence. INPH is progressive (Andrén *et al*, 2014), still probably underdiagnosed (Williams *et al*, 2019) but potentially treatable by CSF diversion (Kazui *et al*, 2015). Familial aggregation is a strong indicator of genetic regulation in the disease process iNPH (Fig 1). Enlargement of brain ventricles is associated with failed cerebrospinal (CSF) homeostasis by so far mostly unknown mechanisms. A mutation of the cilia gene *CFAP43* in iNPH family, confirmed by a knocked‐out mouse model (Morimoto *et al*, 2019), allelic variation of *NME8* (Huovinen *et al*, 2017), a segmental copy number loss in *SFMBT1* in selected iNPH patients (Sato *et al*, 2016), and current results by Yang *et al* (2021) indicate that cilia dysfunction is one of the key mechanisms behind iNPH.

## Graphical abstract

T. Kuulasmaa, M. Hiltunen and V. Leinonen discuss novel genetic and functional findings related to two loss of function deletions in CWH43 gene in patients with Idiopathic normal pressure hydrocephalus by M. Johnson and colleagues, in this issue of *EMBO Mol Med*.

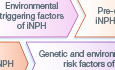

In this issue of *EMBO Molecular Medicine*, Yang *et al* (2021) report novel genetic and functional findings related to two loss of function deletions in *CWH43* gene in patients with iNPH. This is one of the most important studies in the field of iNPH research so far, emphasizing the fact that eventually iNPH is not anymore considered as “idiopathic”. Furthermore, these results strongly support the existence of iNPH as an independent disease entity.

**Figure 1:**
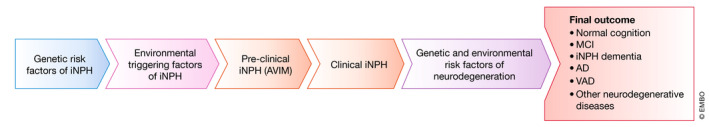
**Hypothetical disease progress of iNPH**

The discovery cohort used in the study of Yang *et al* is relatively small and thus, genetic studies in the larger patient cohorts are expected to identify new potential genetic loci related to iNPH. As with numerous other multifactorial diseases, international collaboration with large multicenter cohorts are needed to uncover the expected rare variants with large effect and more common variants with small effect.

Three out of the eight patients with *CWH43* alteration had potential iNPH‐related family history, indicating that this variant can either be *de novo* mutation or that the familiar inheritance is underestimated due to the late onset of the clinical symptoms. Diagnostic criteria of iNPH need to be updated to include also prodromal iNPH, such as asymptomatic ventriculomegaly with features of iNPH on magnetic resonance imaging (AVIM, Kimihira *et al*, 2020). Identification of genetic variants similar to that now observed with *CWH43* will open a window to decipher the underlying pathophysiological mechanisms further, paving the way to better treatments and novel prevention strategies in the future.
